# Digital finance and climate risk information disclosure

**DOI:** 10.1371/journal.pone.0340383

**Published:** 2026-01-06

**Authors:** Hang Ren, Jianzhong Huang, Jinxin Ren

**Affiliations:** 1 School of Economics, Shanghai University, Shanghai, China; 2 School of International Business, Shanghai University of International Business and Economics, Shanghai, China; University of Almeria: Universidad de Almeria, SPAIN

## Abstract

Under the dual drivers of China’s “Dual Carbon” goals (carbon peaking and carbon neutrality) and the digital technology revolution, the strategic value of corporate climate risk information disclosure has become increasingly prominent. Against this backdrop, this paper systematically explores the relationship between digital finance development and corporate climate risk disclosure using a sample of Chinese A-share listed firms. The research demonstrates that digital finance development significantly promotes corporate climate risk disclosure, a conclusion that remains valid after multiple robustness tests. The study also reveals that digital finance drives climate risk disclosure through alleviating financing constraints, strengthening environmental responsibility, and enhancing reputational incentives. Further analysis indicates that institutional pressure positively moderates the relationship between digital finance and corporate climate risk disclosure; moreover, the impact of digital finance on corporate climate risk disclosure exhibits significant heterogeneity depending on regional factors (rule of law environments and the supply level of digital economy policies), and corporate characteristics (top management backgrounds, lifecycle stages, and equity nature). These findings provide theoretical references and empirical support for balancing digital finance innovation and climate risk governance.

## 1. Introduction

Global climate change has evolved from an environmental issue into a systemic economic risk [1,2]. The 20th edition of the Global Risks Report by the World Economic Forum points out that extreme weather events are undoubtedly one of the most urgent risks in the immediate, short-term, and even long-term. The United Nations Intergovernmental Panel on Climate Change’s (IPCC) Sixth Assessment Report (AR6) also indicates that the climate system has undergone substantial changes on both spatial and temporal scales. As global extreme weather events have become more frequent, the impact of climate risks on economic development has attracted widespread attention. Given that climate risks are global, long-term, and often irreversible, a comprehensive assessment of climate-related risks and opportunities, along with prevention of “green swan” events, critically depends on corporate climate risk information disclosure [3,4].

In recent years, climate risk-related information disclosure has gradually become an important component of global capital market infrastructure construction. Starting from the 2025 fiscal year, the Hong Kong Stock Exchange requires all issuers to disclose and submit climate risk disclosure information that meets the requirements of the International Sustainability Standards Board (ISSB). In April 2025, the Chinese Ministry of Finance, in conjunction with the Ministry of Ecology and Environment, released the “*Guidelines for Sustainable Disclosure of Enterprises No. 1- Climate (Trial) (Draft for Comments)*”, which clarifies the standards and processes for enterprises to provide climate-related information to investors, creditors, the government and its relevant departments, and other stakeholders, facilitating their economic decision-making. The People’s Bank of China has required financial institutions to incorporate climate risk into their comprehensive risk management frameworks, explicitly embedding climate risk factors throughout the entire investment and financing process, and conducting climate risk stress tests, aiming to reduce false advertisements by businesses and bolster investor confidence. In this context, corporate climate risk information disclosure serves not merely as passive compliance with regulatory requirements and the expectations of various parties, but also as an active governance tool that supports the development of climate policies for each region and provides forward-looking evidence for economic operations and the growth of capital markets, thereby making its strategic value increasingly prominent.

However, a fundamental challenge stems from the unique attributes of climate risk information that distinguish it from traditional financial information: First, it is highly forward-looking and uncertain, making it difficult to quantify precisely with specific financial data. Second, it possesses strong externalities and public good attributes, meaning that the private benefits for firms from disclosing climate risk information are often lower than the social benefits, resulting in insufficient endogenous incentives for voluntary disclosure. Third, the measurement metrics and disclosure frameworks lack uniformity, leading to pronounced problems of information non-standardization, which significantly increases the costs and barriers to disclosure [3,4]. These distinctive attributes imply that the primary bottleneck in climate risk information disclosure is not a “pricing bias” of available information in the market, but rather an “insufficient supply” of reliable information.

More critically, the current state of climate risk disclosure in China remains in its infancy, with voluntary disclosure mechanisms remaining dominant. This grants management broad disclosure autonomy, which may lead to selective disclosure of positive information and concealment of negative risks for the purpose of “beautifying performance” or “avoiding negative evaluations” [5]. This behavior further exacerbates the twin challenges of “insufficient supply” and “information distortion.” This reality aligns with the “inefficient equilibrium” described in classical disclosure theory: as Admati and Pfleiderer [6] demonstrate in their cost-benefit model, the optimal disclosure level for “high-cost, low-value” information approaches zero. Unless this cost-benefit structure is fundamentally altered, even a future shift toward mandatory disclosure may repeat the “offset effect” identified by Nejadmalayeri et al. [7], in which high compliance costs erode or even completely nullify the market benefits derived from greater information transparency. This serves as a warning that to address the predicament of climate risk information disclosure necessitates a dual strategy: effectively reducing compliance costs while substantially enhancing the benefits of disclosure.

Digital finance, as a technology-driven revolution in the financial paradigm, with its core characteristics of sharing, convenience, inclusiveness, and low cost, provides a new pathway to address the predicament of climate risk information disclosure. However, the application of digital technology in financial scenarios exhibits a profound “technological duality”: while algorithmic trading (AT) can optimize the transmission efficiency of existing information to asset prices through high-frequency pricing, it may suppress market participants’ initiative to acquire new information by compressing the space for information rents [8]. In contrast, machine learning, leveraging its ability to efficiently process high-dimensional and unstructured data, excels at screening, extracting, and integrating valid information from massive and complex datasets, reducing information noise and bias [9], thereby providing critical support for decision-making related to financial instruments such as green credit and green bonds. The root cause of this seemingly contradictory “technological paradox” lies in the differentiated operational logics of digital technologies within the information ecosystem: AT focuses on the “transaction-based pricing” of information, aiming to optimize the transmission efficiency of existing information, whereas ML concentrates on the “production and extraction” of information, fundamentally enhancing the capacity for new information supply.

Consequently, the net effect of digital finance on climate risk information disclosure is highly dependent on the focus of technological resource application. Should technological resources become excessively skewed toward algorithmic trading on the transaction side, the pursuit of short-term arbitrage gains may crowd out investment in information production. For instance, if financial institutions show greater enthusiasm for short-term arbitrage surrounding green assets rather than supporting enterprises in building long-term disclosure capacity, this would dampen corporate motivation to provide comprehensive climate risk information. Conversely, if priority is given to enhancing information production capabilities through digital technologies like machine learning, corporate willingness to disclose can be strengthened through synergistic effects from technology-driven cost reduction, benefit incentives, and regulatory constraints. For example, firms can efficiently complete the accounting and compilation of climate risk information by leveraging digital technology tools such as machine learning, thereby obtaining benefits such as preferential interest rates on green credit provided by financial institutions. Coupled with the dynamic verification of disclosure quality by digital finance supervision, their willingness and ability to disclose will be systematically improved.

The inherently unstructured nature and high-cost characteristics of climate risk information determine that the key to resolving the challenge of inadequate disclosure lies in the “information production side” rather than the “transaction pricing side.” Only by addressing the core bottlenecks of “difficult production, high costs, and low quality” at the source can corporate willingness to disclose be fundamentally stimulated. Therefore, the theoretical framework of this study is rooted in the “information production side,” focusing on examining how digital finance, through empowerment by digital technologies, gives full play to its core advantages in information processing, resource allocation, and regulatory effectiveness, thereby encouraging enterprises to proactively produce and disclose high-quality climate risk information. This approach not only offers a novel theoretical perspective and practical pathway to tackle the current challenge of inadequate climate risk information disclosure but also holds significant theoretical and practical implications for facilitating the “capital-climate” information cycle and guiding financial resources to provide targeted support for the green transition in the context of the “Dual Carbon” strategy.

Existing research provides an important foundation for this study. Scholars have explored the factors influencing climate risk information disclosure from perspectives such as firm characteristics [10,11] and external environment [12–14]. Meanwhile, extensive studies have demonstrated that digital finance exerts significant and far-reaching impacts on both the macroeconomic system [15–19] and micro-market entities [20,21]. However, few studies have integrated digital finance and climate risk information disclosure into a unified analytical framework, nor have they systematically elucidated how digital finance can overcome the theoretical dilemma of “high costs leading to insufficient disclosure” as revealed by Admati and Pfleiderer [6]. Furthermore, although some studies touch upon institutional factors [12], they merely identify them as influencing factors of climate risk information disclosure without delving into the moderating role of institutional pressures (e.g., government environmental attention, media supervision) in the “digital finance-disclosure relationship.” Consequently, these studies fail to address the practical concern of “whether the effects of digital finance vary across different contexts.”

Based on this, utilizing a sample of China’s A-share listed enterprises from 2016 to 2023, this study systematically examines the driving effect of digital finance on corporate climate risk information disclosure. The marginal contributions of this research may be threefold: (1) It expands the research on the economic consequences of digital finance. Moving beyond traditional research boundaries, this study extends the perspective to the field of “climate risk information disclosure,” revealing the intrinsic logic of how digital finance drives climate disclosure through three core mechanisms: “financing constraint alleviation, enhanced environmental responsibility, and reputational incentives.” This provides new empirical evidence and theoretical explanations for the interdisciplinary field of “digital finance and climate governance.” (2) It enriches the research system on factors influencing corporate climate risk information disclosure. Differing from existing studies that often focus on firm-level micro-characteristics or the macro-environment, this study constructs an influence chain from regional digital finance—a perspective characterized by strong externalities and technological empowerment—to corporate disclosure behavior, and further introduces the moderating effect of institutional pressure on this influence chain’s role, providing a systematic explanation for understanding corporate non-financial information disclosure practices. (3) It enhances the policy adaptability of the research findings. The study systematically analyzes the heterogeneous effects of digital finance on climate risk information disclosure by innovatively integrating firms’ external environments and internal characteristics, thereby providing a scientific basis and practical guidance for policy design under different contexts.

## 2. Theoretical analysis and research hypotheses

### 2.1. Digital finance and corporate climate risk information disclosure

Dynamic capability theory posits that firms must continuously adjust their resources and capabilities to adapt to external environmental changes in order to build a sustainable competitive advantage [22,23]. Against the backdrop of the comprehensive advancement of current green development strategies, corporate disclosure of climate risk—a quintessential systemic environmental shock—serves not merely as a compliance requirement to respond to regulations and stakeholder demands, but also as a crucial strategic choice for obtaining green financial resources and shaping low-carbon competitiveness. However, constrained by inherent limitations such as information asymmetry and financial inefficiencies, the traditional financial system struggles to accurately assess corporate climate performance, leading to resource misallocation. This significantly dampens corporate willingness to disclose climate risk information and exacerbates the practical challenge of inadequate disclosure. Within this context, digital finance, a new paradigm of deep integration between digital technology and financial innovation, leverages its core advantages in information processing, resource allocation, and regulatory effectiveness to provide a viable pathway to address this dilemma, thereby increasingly emerging as a key driver of corporate climate risk information disclosure.

First, digital finance is reshaping the generation and verification processes of climate risk information, thereby overcoming the “information supply” bottleneck. Leveraging big data and artificial intelligence (AI) models, it transforms forward-looking, unstructured, and ambiguous climate risks into quantifiable, analyzable structured metrics [24], effectively solving the “quantification challenge.” Employing technologies such as Natural Language Processing (NLP) to automate the extraction and integration of key information and promote the standardization of disclosure formats, it significantly reduces the costs associated with information processing, compilation, and verification [25]. Furthermore, utilizing blockchain’s immutability and traceability features, it provides inherent credibility assurance for disclosed information, alleviating information asymmetry at the source and preventing the emergence of a “*lemons market”* effect [26]. Second, digital finance restructures the incentive-compatibility mechanism for resource allocation, effectively resolving the “insufficient motivation” dilemma. Leveraging dynamic credit assessment models, digital finance platforms directly link the quality of climate risk information disclosure to green credit lines and interest rate pricing, enabling companies with robust disclosures to access lower-cost funding [13]. Simultaneously, through tools such as robo-advisors and green financial instruments, they channel social capital towards high-transparency enterprises, creating a virtuous cycle of “disclosure – capital inflow – enhanced competitiveness.” This process translates climate performance into tangible financial and competitive advantages for firms, thereby resolving the “incentive inadequacy” dilemma arising from “strong externalities.” Finally, the RegTech (Regulatory Technology) facilitated by digital finance significantly enhances financial regulators’ capacity to identify and monitor climate risks, establishing a “regulatory constraint.” Regulatory authorities can utilize big data and cloud computing technologies to build a unified national corporate environmental information database, drastically improving the efficiency of data collection and identification. Moreover, through the application of AI, blockchain, and the Internet of Things (IoT) technologies, they can achieve real-time scrutiny and dynamic monitoring of corporate climate risk management. This normalized, penetrating regulatory system substantially increases the expected cost for firms concealing climate risks (based on Deterrence theory [27]). To avoid potential compliance penalties and reputational damage, companies are compelled to integrate climate risk management into their core governance frameworks and continuously improve their information disclosure quality. Therefore, digital finance does not merely unidirectionally increase or decrease a specific cost of corporate climate risk information disclosure. Instead, through the synergistic effect of technological empowerment, market incentives, and regulatory constraints, it systematically reshapes the decision-making framework for corporate climate risk information disclosure, transforming it from a “high-cost, low-return” compliance burden into a “sustainable, rewarding” strategic investment.

Based on this, the following hypothesis is proposed

H1: The development of digital finance can drive companies to disclose climate risk information.

### 2.2. The mechanism of digital finance on corporate climate risk information disclosure

Existing research indicates that corporate decisions regarding climate risk information disclosure are shaped by the trade-offs among three key factors: the financing constraints faced by the firm, which determine its capacity to bear the associated costs of disclosure; the environmental responsibilities it must fulfill, which reflect the compliance pressure from regulatory requirements and societal expectations; and the incentives arising from reputational enhancement, which pertain to the market trust and competitive advantages gained through disclosure. Building on this foundation, this study analyzes three core mechanisms: the financing constraint alleviation effect, the environmental responsibility fulfillment effect, and the reputational incentive effect.

First, digital finance can drive corporate climate risk information disclosure by alleviating financing constraints. The Resource-Based View posits that financial resource endowments constitute a fundamental constraint on firms’ implementation of strategic activities. As a non-mandatory yet long-term strategic management practice, climate risk information disclosure requires sustained investment of specialized financial and human resources for data collection, accounting, and verification. When firms face severe financing constraints, management tends to prioritize allocating scarce resources to maintaining short-term operations and core business activities, while relegating long-term investments with public-good attributes—such as climate risk management—to a secondary position. This creates a critical bottleneck that inhibits high-quality climate risk information disclosure [28]. The rise of digital finance offers a potential solution to this bottleneck. Specifically, First, digital finance leverages the advantages of digital technology to innovate financial products, thereby overcoming the limitations of traditional credit models that rely heavily on collateral. This transformation facilitates the construction of diversified financing networks, effectively broadening firms’ financing channels. Second, digital finance empowers the optimization of traditional financial services processes, substantially reducing financing time and improving the financing efficiency of enterprises. Third, financial institutions utilize digital technology to construct dynamic credit assessment models by deeply integrating multi-dimensional data resources, enabling comprehensive and objective evaluation of corporate creditworthiness and effectively breaking down information barriers in financial markets, which significantly enhances firms’ access to credit. The alleviation of financing constraints provides essential resource support and strengthens disclosure incentives. On the one hand, the reduction in financial pressure enables firms to reorient their strategic focus toward environmental management systems—including hiring specialists, deploying monitoring systems, and improving data governance—thereby enhancing the accuracy and completeness of climate risk information disclosure data. On the other hand, improved financing conditions enhance firms’ ability to meet the eligibility criteria for green finance. As the green financial system deepens, environmental performance is increasingly incorporated into credit assessments by financial institutions. To fulfill the approval requirements for green financial products and secure lower-cost funding, firms are motivated to proactively disclose climate risk information [13,29]. In summary, by alleviating corporate financing constraints, digital finance not only enhances firms’ capacity for climate risk information disclosure but also fundamentally strengthens their motivation to disclose.

Second, digital finance strengthens corporate assumption of environmental responsibility to drive climate risk information disclosure. Legitimacy theory posits that an organization’s survival and development depend on the alignment of its behaviors with societal values and expectations—that is, on obtaining and maintaining “legitimacy” [30]. Against the backdrop of green development strategies becoming a broad social consensus, corporate fulfillment of environmental responsibilities constitutes a key manifestation of their commitment to social responsibility and acquisition of legitimacy. However, information asymmetry creates opportunities for enterprises to conceal environmental violations, leading to the failure of external supervision. This severely impedes the market’s accurate understanding of corporate environmental performance and thereby weakens the external pressure and internal motivation for enterprises to fulfill their environmental responsibilities [31]. The development of digital finance effectively addresses this dilemma. Specifically, the technological innovation advantages of digital finance enable the precise capture of corporate environmental information, particularly in exposing previously hidden environmental violations [32], thereby significantly enhancing information transparency and compelling firms to assume environmental responsibilities. Simultaneously, by constructing a supply chain finance ecosystem, digital finance accelerates the formation of communities of shared interest among upstream and downstream firms in the industrial chain. Within this structure, environmental demands from the investment side (e.g., creditors’ environmental investment constraints) and the consumption side (e.g., consumers’ environmental preferences) can be transmitted along the supply chain, further urging firms to fulfill environmental responsibilities. Moreover, financial institutions leverage digital finance tools to establish environmental value assessment systems, incorporating climate risks as a core dimension in financial resource allocation. By implementing mechanisms such as differentiated pricing of green credit based on corporate environmental performance, they achieve dynamic and precise capital allocation, further guiding firms to fulfill their environmental responsibilities. Therefore, under the multidimensional pressure intensified by digital finance, proactive disclosure of climate risk information has become a strategic tool for firms to actively seek social legitimacy by demonstrating their environmental accountability. Existing research also provides robust evidence that firms actively fulfilling environmental responsibilities tend to engage in higher-quality and more substantive environmental information disclosure to demonstrate the compliance and strategic foresight of their actions to stakeholders [33–35].

Finally, digital finance provides intrinsic incentives for corporate climate risk information disclosure by building and enhancing corporate reputation. Corporate reputation, formed through long-term interactions with various stakeholders, refers to an overall evaluation of a firm’s capabilities, credibility, and sense of responsibility. It is a difficult-to-imitate strategic asset [36] that essentially embodies social trust in and recognition of the firm, serving as a source of competitive advantage. With the accelerating transparency of environmental information, reputation management has become a key driver for firms choosing to disclose climate risk information [4,37]. The emergence of digital finance is profoundly transforming the logic of building and maintaining corporate reputation by reshaping how information diffuses through social networks. On the one hand, digital finance builds corporate reputation by enhancing information credibility. Digital financial infrastructure based on technologies like blockchain ensures the immutability, traceability, and verifiability of corporate information disclosure [38]. This provides strong technical endorsement for corporate disclosed information, significantly reducing stakeholders’ verification costs, thereby laying a solid foundation for building a transparent and reliable reputation. Simultaneously, this trust mechanism maintains consumer brand loyalty and supplier collaboration stickiness, stabilizes the core relationship network for corporate value creation, ensures sustained and stable cash flow, reduces the likelihood of corporate financial distress, and thus provides a stable foundation for the long-term accumulation of reputational capital [39,40]. On the other hand, digital finance safeguards corporate reputation through its risk early-warning function. The green financial risk control systems established by financial institutions using fintech can effectively identify potential environmental compliance risks, which is crucial for warning against and avoiding the devastating reputational damage from environmental scandals, as highlighted in research by Hasan et al. [41]. Under this dual mechanism of “construction and defense,” reputational capital is accumulated and enhanced, becoming a key driver for corporate climate risk information disclosure. According to Signaling theory, having established a valuable green reputation, firms are motivated to conduct more comprehensive and higher-quality climate risk information disclosure [37] to send positive signals to the market that they outperform their competitors [42]. In the digital finance ecosystem, such disclosure signals can achieve efficient diffusion through social networks, accelerating market recognition of corporate reputation and transforming it into tangible financing advantages [43], thereby providing strong intrinsic economic incentives for disclosure. Conversely, if firms still choose to disclose passively or conceal climate risks in the high-transparency environment brought about by digital finance, such negative behavior could rapidly diffuse through social networks, triggering a crisis of trust and causing significant reputational damage.

Based on this, the following hypothesis is proposed:

H2: Digital finance can promote corporate climate risk information disclosure by alleviating financing constraints, strengthening environmental responsibility, and enhancing corporate reputation.

## 3. The design for research

### 3.1. Sample selection and data sources

Considering the availability and continuity of data, this study selects Chinese A-share listed companies spanning the period from 2016 to 2023 as the research sample. Among them, the digital finance index is derived from *the Peking University Digital Inclusive Finance Index: 2011–2023*. The basic information and financial data of the firm are sourced from *the China Economic and Financial Research Database (CSMAR)*. The foundational data for cities is compiled from multiple sources, including *The Statistical Yearbook of China’s Cities*, *The Statistical Yearbook of Urban Construction in China*, *annual statistical yearbooks* of each province (and city), *Statistical Announcements on the National Economy and Social Development* of each city, government websites, and the Eps City Database. All other data mentioned in the passage comes from *the China Research Data Service Platform (CNRDS)* and the *China Urban Business Environment Database.* At the same time, in order to enhance the reliability of the data, samples from the financial industry, ST, *ST, and PT companies were excluded during data processing, and samples with missing data were also omitted. Ultimately, 26365 valid firm-year observations were obtained for analysis.

### 3.2. Model settings

In order to identify the impact of digital finance development on corporate climate risk information disclosure, the following model is set up for empirical testing:


$$Y_{it}=\alpha_{\text{0}}+\alpha_{\text{1}}df_{it}+\alpha_{\text{2}}{Controls}_{it}+\mu_{t}+\lambda_{i}+\delta_{j}+\varepsilon_{it}\;$$ (1)

Among them, the subscripts *i* and *t* represent enterprise and time, respectively. $Y_{it}$ is the dependent variable, representing the degree of climate risk information disclosure of company *i* in year *t*. $df_{it}$ is the core explanatory variable, signifying the level of urban digi*t*al finance development in the city where enterprise *i* is located in year *t*. ${Controls}_{it}\;$is a series of control variables. $\mu_{t}$ represents the time fixed effect, $\lambda_{i}$ denotes the individual fixed effec*t* of the enterprise, $\delta_{j}$ is the industry fixed effect, and $\varepsilon_{it}$ is the random perturbation term. In Model (1), if $\alpha_{\text{1}}$ is significantly positive, it indicates that the development of digital finance can drive corporate climate risk information disclosure.

### 3.3. Variables

#### 3.3.1. Dependent variable.

Climate Risk Information Disclosure (*CRID*). Given the absence of mandatory climate change information disclosure requirements, there is currently no direct quantitative indicator that can measure the degree of corporate climate change risk disclosure. Therefore, this study employs text analysis to construct proxy variables for climate risk information disclosure, addressing the absence of direct quantitative indicators under voluntary disclosure regimes. At present, in the field of text analysis, word frequency analysis is widely adopted as a core method to quantify the degree of attention of a text to specific semantic categories, but this approach is easily affected by the length of the text. To address this limitation, this study uses the term frequency ratio method, which calculates the proportion of target vocabulary frequency to the total number of words in the text, effectively mitigating the impact of text size differences on semantic strength measurement. Based on this, this study constructs the corporate climate risk disclosure index by using the ratio of the climate risk-related vocabulary to the total number of words in the text as a proxy variable. Specifically, textual analysis and machine learning methods are used here to construct corporate climate risk disclosure indicators. First, drawing on the research of Lin and Wu [44], the method of “*seed word set + similar word expansion*” is adopted to screen out keywords with a semantic similarity of more than 0.5 to “climate risk”. Subsequently, interference from incorrectly expressed words, neutral terms, meaningless vocabulary, etc., was removed, ultimately obtaining the final set of keywords related to climate risk information, as detailed in Table 1. Finally, the extent of a firm’s climate risk information disclosure is measured by the ratio of the total frequency of the aforementioned keywords in the annual report to the total word count of the report, multiplied by 100. A higher value of this indicator denotes a greater level of climate risk information disclosure by the firm.

**Table 1:** Corporate climate risk information disclosure keyword set.

Corporate climate risk information	
keyword set	New energy, Electric vehicles, Wind energy, Hydropower, Nuclear energy, Solar energy, Photovoltaics, Wind power generation, Hydropower, Nuclear power, Hydrogen energy, Energy storage, Carbon sink, forest, Ocean, Technology, Innovation, Battery, Infrastructure, Energy efficiency, Carbon neutrality, Carbon peak, Emissions reduction, Carbon trading market, Climate change, Ecology, Air temperature, Precipitation, Drought, Flood disaster, Natural disaster, Pollution prevention and control, Waste gas, Wastewater, Dust, Cleaning, Low-carbon, Energy saving, Environmental protection, High-efficiency, Optimization, New-type, Industrial structure, Energy structure, Green, Research and development, Green bond, Ammonia nitrogen, Ammonia gas, Protect the environment, Heavy rain, Extreme sun exposure, Muggy, Warming, Charging, Odor, Store energy, Standard-compliant discharge, Atmosphere, Heavy fog, Rainwater, Nitrogen oxides, Ground temperature, Low energy consumption, Electric car, Electric power, Power battery, Power system, Freezing rain, Rainy, Sulfur dioxide, Power generation, Waste, Wastewater treatment, Fine particulates, Wind power, Wind sensation, Wind force, Fluorides, High energy consumption, Energy-intensive operations, Industrial wastewater, Solid waste, Solid discarded materials, Energy consumption, Flooding, Environmentally friendly, Annular pressure, Carbon mitigation, Consumption reduction, Carbon reduction, Snowfall, Rainfall, Energy saving and consumption reduction, Energy conservation, Conservation-oriented, Resource conservation, Slagging, Particulate matter, Renewable, Zero emission, Zero-carbon, Zero carbon emissions, Hydrogen sulfide, Hydrogen chloride, Energy consumption, Energy efficiency, Energy, Inverter, Emission discharge, Total discharge emission, Oxygen discharge, Climate, Meteorological elements, Heavy rainfall, Hydrogen energy, Fuel cell, Thermal diffusion, Three wastes, Heat dissipation, Process wastewater, Dual carbon goals, Acid mist, Carbon peak, Weather, Outflow discharge, Tail gas exhaust, Pollution, Sewage, Dust-free, Infiltration, Smoke flue dust, Pollution control, Heavy metals, Resource-saving, Total nitrogen, and Dust holding capacity.

#### 3.3.2. Core explanatory variable.

Digital finance (*df*). Urban digital finance development level is measured using the natural logarithm of the Peking University Digital Inclusive Finance Index, which comprehensively evaluates the development of urban digital finance from coverage breadth, usage depth, and digitization degree.

#### 3.3.3. Control variables.

To build a reliable econometric model and minimize omitted variable bias, the selection of control variables in this study strictly adheres to a triple principle: theoretical relevance, established conventions in the existing literature, and data availability. Theoretically, Legitimacy theory [30] posits that firms engage in disclosure to respond to external pressures and maintain operational legitimacy; Stakeholder theory [45] indicates that corporate disclosure decisions are constrained by firms’ internal resources and capabilities. Building on this, and to concurrently capture the joint influence of external pressure and internal capacity on corporate climate risk information disclosure while controlling for other potential confounding factors, we draw upon existing studies to select control variables from both the firm micro-level and the city macro-level [11,28,46,47]. The firm-level control variables include the following: Operating profit margin (*opm*) is measured as the ratio of operating profit to operating revenue, reflecting a firm’s profitability. Firms with stronger profitability are generally considered to possess more ample resources to invest in environmental management and disclosure. Leverage ratio (*lev*) is represented by the ratio of total liabilities to total assets, gauging a firm’s long-term solvency and financial risk. Firms with higher financial leverage may face different disclosure motivations and pressures. Current ratio (*curr*) is measured as the ratio of current assets to current liabilities, assessing a firm’s short-term solvency. Firms with stronger short-term solvency possess greater financial slack, potentially leading to a stronger willingness to allocate resources to non-urgent activities like information disclosure; conversely, firms under high short-term debt repayment pressure may prioritize securing working capital, reducing disclosure investments. Development capability (*tagr*) is captured by the year-on-year growth rate of total assets. Firms in a high-growth phase typically place greater emphasis on long-term reputation building and may engage in information disclosure more proactively. The city-level control variables include the following: Industrial structure advancement (*ins*) is measured as the ratio of the value-added of the tertiary industry to that of the secondary industry, to control for the impact of regional industrial structure. Regions with a more advanced industrial structure often have stricter environmental regulations, greater penetration of green finance concepts, and more attentive stakeholders regarding corporate environmental performance, which may exert pressure on firms to disclose climate risk information. Population density (*pd*) is represented by the density of the permanent population. Densely populated areas are often characterized by greater public opinion pressure and more intense media scrutiny, potentially subjecting firms to higher societal monitoring pressure and thus incentivizing proactive environmental disclosure [48]. Infrastructure level (*road*) is quantified by per capita road area. Well-developed infrastructure facilitates resource flows and can reduce firm operating costs, thereby influencing corporate disclosure behavior [49]. Informatization technology level (*phone*) is characterized by the year-end number of mobile phone subscribers in each region, reflecting the efficiency and transparency of information dissemination within a city. In cities with higher informatization levels, the cost for stakeholders to access corporate information is often lower, thereby reducing informational asymmetries for firms regarding environmental disclosure and prompting them to improve disclosure quality to avoid trust crises arising from information asymmetry [24]. Data for these variables were sourced from the *CSMAR database*, the *China City Statistical Yearbook*, and the *Eps City Database*. The descriptive statistics for the main variables are presented in Table 2.

**Table 2:** Descriptive statistics.

Variable	N	Mean	SD	Min	Max
CRID	26365	0.927	0.703	0.000	9.726
df	26365	5.669	0.154	5.121	5.922
lev	26365	0.418	0.207	0.008	1.957
curr	26365	2.625	3.174	0.028	80.664
opm	26365	0.048	0.887	−95.186	14.043
tagr	26365	0.192	0.603	−0.929	37.029
ins	26365	1.891	1.215	0.333	6.387
pd	26365	0.193	0.227	0.001	0.895
road	26365	0.168	0.082	0.023	0.576
phone	26365	17.957	13.269	0.320	46.918

## 4. Empirical result analysis

### 4.1. Baseline regression results

We begin by estimating Model (1) to examine the relationship between digital finance development and corporate climate risk information disclosure. The regression results are shown in Table 3. Among them, column (1) is the regression result of ordinary OLS estimation, which preliminarily verifies the relationship between digital finance and corporate climate risk information disclosure. Column (2) shows the estimated results of the two-way fixed effects model with the addition of individual fixed effects and time fixed effects, while column (3) shows the regression results with the further addition of industry fixed effects. It can be seen that regardless of whether fixed effects are controlled for, the estimated coefficients of digital finance are significantly positive, indicating that digital finance can promote firms to disclose climate risk information, thus supporting hypothesis H1.

**Table 3:** The result of baseline regression.

Variable	(1)	(2)	(3)
	CRID	CRID	CRID
df	0.571***	0.488**	0.505***
	(0.032)	(0.189)	(0.137)
lev	0.344***	0.088**	0.084***
	(0.027)	(0.036)	(0.024)
curr	−0.009***	−0.002*	−0.002**
	(0.001)	(0.001)	(0.001)
opm	0.010**	0.005*	0.005
	(0.005)	(0.003)	(0.003)
tagr	0.042***	0.011***	0.011***
	(0.010)	(0.003)	(0.004)
ins	0.016**	0.012	0.011
	(0.007)	(0.013)	(0.010)
pd	0.005	−0.320***	−0.280***
	(0.028)	(0.097)	(0.076)
road	0.503***	0.021	0.035
	(0.078)	(0.090)	(0.067)
phone	−0.001	−0.001	−0.001
	(0.001)	(0.001)	(0.001)
Constant	−2.545***	−1.883*	−1.915**
	(0.171)	(1.013)	(0.769)
Firm FE	N	Y	Y
Year FE	N	Y	Y
Industry FE	N	N	Y
Observations	26,365	26,365	26,365
R-squared	0.033	0.189	0.891

Note: ***, **, and * represent statistical significance levels of 1%, 5%, and 10%, respectively. Robust standard errors are reported in parentheses, the same applies below.

### 4.2. Robustness test

#### 4.2.1. Replace variables.

First, we replace the dependent variable. Drawing on the climate risk vocabulary set constructed by Du J. et al. [50], we remeasure the level of corporate climate risk information disclosure by applying the keyword set presented in Table 4. The regression results, presented in column (1) of Table 5, show that the estimated coefficient on the development of digital finance remains significantly positive. Second, we replace the core explanatory variable. We employ the number of fintech companies in each city as an alternative proxy for the level of digital finance development in cities and conduct regression analysis again. The results, presented in column (2) of Table 5, demonstrate that the estimated coefficient on the core explanatory variable remains significantly positive. These findings confirm that the core conclusion that digital finance development can improve the level of corporate climate risk information disclosure remains robust, and this positive effect is not affected by the variable measurement methods.

**Table 4:** Corporate climate risk information disclosure keyword set.

Corporate climate risk information	
keyword set	Energy conservation; Energy; Cleaning; Ecology; Environment; Transformation; Solar energy; Upgrading; Circularity; Utilization rate; Nuclear power; Wind power; Natural gas; Efficiency enhancement; Fuel oil; Efficiency; Renewable; Emission reduction; Environmental protection; Green; Low-carbon; Consumption reduction; Fuel; Water conservation; Photovoltaics; High-efficiency; Retrofit; Fuel consumption; Electricity consumption; Energy consumption; Wind power; Photovoltaics; Performance; Intensive; Disaster; Earthquake; Typhoon; Tsunami; Drought-flood; Extreme; Adverse; Urban flooding; Gale; Sandstorm; Hurricane; Frost; Flood disaster; Storm; Debris flow; Landslide; Ice-jam freezing; Snow disaster; Drought disaster; Flooding; Rainstorm; Tornado; Hail; Flood disaster; Rain-snow; Ice freeze; Blizzard; Freeze damage; Drought; Drought conditions; Heavy rainfall; Flood; Severe cold; Sand wind; Climate; Weather; Humidity; Water temperature; Cooling; Cold; Air temperature; Rainfall; Temperature; Rainwater; Rainy season; Rainfall conditions; Precipitation; Overcast rain; Rainy; Extreme cold; Winter; Flood season; High humidity; Water conditions; Water level; Sunlight; Water shortage; Alpine cold; Caold wave; Settlement; Groundwater; Flood situation; Land surface; Water storage.

**Table 5:** The results of robustness tests (i).

	Replace variables		Add fixed effects	Exclude interfering factors	
Variable	(1)	(2)	(3)	(4)	(5)
	CRID	CRID	CRID	CRID	CRID
df	0.176**		0.511***	0.435***	0.603***
	(0.075)		(0.154)	(0.149)	(0.146)
fintech		0.040***			
		(0.006)			
lev	0.027**	0.082***	0.076***	0.094***	0.088***
	(0.012)	(0.024)	(0.024)	(0.026)	(0.026)
curr	−0.001*	−0.002**	−0.002**	−0.003**	−0.002
	(0.000)	(0.001)	(0.001)	(0.001)	(0.001)
opm	0.002	0.005	0.004	0.004	0.006
	(0.002)	(0.003)	(0.003)	(0.005)	(0.004)
tagr	0.003**	0.011***	0.011***	0.010***	0.015***
	(0.002)	(0.004)	(0.004)	(0.004)	(0.004)
ins	0.004	0.008	0.011	0.007	0.013
	(0.005)	(0.010)	(0.012)	(0.014)	(0.011)
pd	−0.101**	−0.314***	−0.606***	−0.360***	−0.294***
	(0.040)	(0.075)	(0.116)	(0.088)	(0.085)
road	−0.050	0.046	−0.019	0.026	0.062
	(0.035)	(0.067)	(0.070)	(0.069)	(0.074)
phone	−0.000	−0.003***	−0.004***	−0.000	−0.002
	(0.001)	(0.001)	(0.001)	(0.002)	(0.001)
Constant	−0.624	0.778***	−1.823**	−1.512*	−2.476***
	(0.423)	(0.038)	(0.871)	(0.838)	(0.821)
Firm FE	Y	Y	Y	Y	Y
Year FE	Y	Y	Y	Y	Y
Industry FE	Y	Y	Y	Y	Y
City FE	N	N	Y	N	N
Observations	26,365	26,365	26,365	20,996	22,935
R-squared	0.889	0.891	0.893	0.885	0.885

Note: ***, **, and * represent statistical significance levels of 1%, 5%, and 10%, respectively. Robust standard errors are reported in parentheses.

#### 4.2.2. Add fixed effects.

To address potential biases from time-invariant omitted variables at the regional level, such as those resulting from listed firms relocating their registration addresses, the city-level fixed effects are added to Model (1) to control for time-invariant city-level heterogeneity. The regression result, presented in column (3) of Table 5, demonstrates that the estimated coefficient on digital finance remains significantly positive. This finding confirms that the regression result in the previous section is still robust and reliable.

#### 4.2.3. Exclude interfering factors.

On the one hand, given that the special policy environment and economic structure of municipalities directly under the central government in China (Beijing, Shanghai, Tianjin, Chongqing) may bias the regression results, these cities are removed from the sample for a robustness test. The regression results in column (4) of Table 5 show that the estimated coefficient on digital finance is significantly positive. On the other hand, the outbreak of COVID-19 in 2020 introduced substantial disruptions to the business environment, market expectations and policy orientation faced by enterprises, which may alter corporate behaviors and strategies regarding climate risk information disclosure. Therefore, in order to mitigate potential interference from this exogenous shock with the regression results, the 2020 sample observations are excluded and the regression is rerun. Column (5) of Table 5 shows that the digital finance coefficient remains significantly positive. These robustness checks further validate the positive impact of digital finance on corporate climate risk information disclosure.

#### 4.2.4 Adjust abnormal samples.

To mitigate the impact of outliers on the regression results, 1% bilateral winsorization is performed on all continuous variables in the model at this stage, followed by re-estimation of the model. The results are shown in columns (1) of Table 6, and the estimated coefficients on digital finance remain significantly positive, once again verifying the positive correlation between digital finance and corporate climate risk information disclosure.

**Table 6:** The results of robustness tests (ii).

	Winsorization	Lag the Core Explanatory Variable
Variable	(1)	(2)
	CRID	CRID
df	0.422***	
	(0.125)	
L1.df		0.460***
		(0.120)
lev	0.106***	0.083***
	(0.026)	(0.024)
curr	−0.004**	−0.002**
	(0.002)	(0.001)
opm	0.047***	0.005
	(0.011)	(0.003)
tagr	0.022***	0.011***
	(0.006)	(0.004)
ins	0.009	0.008
	(0.010)	(0.010)
pd	−0.263***	−0.279***
	(0.074)	(0.076)
road	0.051	0.046
	(0.067)	(0.067)
phone	−0.001	−0.001
	(0.001)	(0.001)
Constant	−1.471**	−1.634**
	(0.703)	(0.664)
Firm FE	Y	Y
Year FE	Y	Y
Industry FE	Y	Y
Observations	N	N
R-squared	26,365	26,365

Note: ***, **, and * represent statistical significance levels of 1%, 5%, and 10%, respectively. Robust standard errors are reported in parentheses.

#### 4.2.5 Lag the core explanatory variable.

To mitigate potential bias in the regression results due to temporal lag effects, we conduct a robustness test by replacing the core explanatory variable with its one-period lagged value (i.e., substituting the period t value with the period t-1 value). The results, presented in column (2) of Table 6, indicate that lagged digital finance development has a significantly positive effect on corporate climate risk information disclosure in the current year. This finding further validates the robustness of the baseline regression results.

### 4.3. Endogeneity test

Generally speaking, it is difficult for a single firm to influence or alter regional digital finance development. Therefore, this study is relatively less susceptible to endogeneity issues arising from reverse causality. Nonetheless, the model might still suffer from endogeneity problems caused by unobserved omitted variables or measurement errors. On the one hand, although the baseline model already controls for firm, year, and industry fixed effects, and has incorporated key variables at both the firm and city levels as much as possible, it remains impossible to exhaust all potential unobserved factors that are correlated with digital finance development and simultaneously significantly affect corporate climate risk information disclosure. On the other hand, the measure of the corporate climate risk information disclosure level, derived from textual data analysis, may also be subject to measurement errors. To address the potential impact of these two issues on the core findings presented earlier, this paper attempts to employ the instrumental variable approach to mitigate endogeneity concerns.

First, drawing on the approach of Nunn and Qian [51], we construct the first instrumental variable (IV1): the interaction term between the spherical distance from a firm’s located city to Hangzhou and a time trend term. Hangzhou is the technological birthplace and innovation center of digital finance in China. Cities geographically closer to Hangzhou are more likely to benefit from knowledge spillovers and demonstration effects, potentially leading to a higher level of digital finance development in those regions, thus satisfying the relevance condition for the instrument. Simultaneously, the historically determined geographical distance is exogenous and does not directly affect a firm’s climate risk information disclosure, thereby satisfying the exogeneity condition for the instrumental variable. Second, following the methodology of Goldsmith-Pinkham et al. [52], this paper constructs a second instrumental variable (IV2): the interaction term between the base period (t-1) digital finance index of each prefecture-level city and the contemporaneous national growth rate of the digital finance index. This method utilizes a shift-share IV to address endogeneity issues. The underlying logic is to simulate the annual estimated values for each analytical unit using the initial shares (the lagged local index) and an aggregate shock (the national growth rate). These estimates are highly correlated with the actual values but uncorrelated with the residual terms of other variables. Specifically, this instrument is highly correlated with the level of digital finance development at the city level, fulfilling the relevance condition, because a region’s prior digital finance foundation determines its subsequent growth potential. Concurrently, the historical national-level growth rate of digital finance is a macroeconomic aggregate that is exogenous to individual firms and unlikely to directly affect an individual firm’s climate risk information disclosure despite specific city characteristics, thus plausibly satisfying the exogeneity condition. Table 7 reports the estimation results of the instrumental variable approach in detail. Columns (1) and (3) present the first-stage regression results, showing that the coefficients of the instrumental variables are statistically significant, indicating that the instruments satisfy the relevance assumption, as expected. Furthermore, the Kleibergen-Paap rk LM statistics are significant at the 1% level, rejecting the null hypothesis of underidentification. The Kleibergen-Paap rk Wald F statistics are also substantially greater than the Stock-Yogo critical value of 16.38 at the 10% level, indicating the absence of a weak instrument problem. These tests collectively reinforce the validity of the selected instruments. In the second-stage regression results shown in columns (2) and (4), the estimated coefficients for digital finance remain significantly positive. This suggests that the positive promoting effect of digital finance development on corporate climate risk information disclosure holds even after mitigating endogeneity concerns, further corroborating the robustness of the core findings.

**Table 7:** The results of endogeneity tests.

	IV1		IV2	
Variable	(1)	(2)	(3)	(4)
	df	CRID	df	CRID
df		1.744*		1.040***
		(0.938)		(0.346)
IV1	−0.000***			
	(0.000)			
IV2			1.204***	
			(0.035)	
lev	−0.000	0.084***	0.001	0.084***
	(0.001)	(0.024)	(0.001)	(0.024)
curr	0.000*	−0.002**	0.000**	−0.002**
	(0.000)	(0.001)	(0.000)	(0.001)
opm	0.000	0.005	0.000	0.005
	(0.000)	(0.003)	(0.000)	(0.003)
tagr	0.000*	0.011***	0.000*	0.011***
	(0.000)	(0.003)	(0.000)	(0.004)
ins	0.004***	0.007	0.004***	0.009
	(0.001)	(0.011)	(0.001)	(0.010)
pd	0.093***	−0.393***	0.087***	−0.329***
	(0.010)	(0.110)	(0.009)	(0.081)
road	0.054***	−0.038	0.034***	0.004
	(0.005)	(0.089)	(0.005)	(0.070)
phone	0.002***	−0.004*	0.002***	−0.002**
	(0.000)	(0.002)	(0.000)	(0.001)
Firm FE	Y	Y	Y	Y
Year FE	Y	Y	Y	Y
Industry FE	Y	Y	Y	Y
Observations	26,365	26,365	26,365	26,365
R-squared	0.993	−0.001	0.994	0.003
Kleibergen-Paap rk LM statistic	31.886***		1241.447***	
Kleibergen-Paap rk Wald F statistic	28.015 [16.38]		1157.499 [16.38]	

Note: ***, **, and * represent statistical significance levels of 1%, 5%, and 10%, respectively. Robust standard errors are reported in parentheses. Critical value of the Stock-Yogo weak instrumental variable test at the 10% significance level are reported in square brackets.

### 4.4. Mechanism test

As delineated in our theoretical analysis, the alleviation of financing constraints, the fulfillment of environmental responsibility, and the reinforcement of reputational incentives constitute three pivotal channels through which digital finance influences corporate climate risk information disclosure. To empirically test research hypothesis H2, we focus on examining the impact of digital finance on these mechanism variables. The following regression model is constructed:


$$M_{it}=\alpha_{\text{0}}+\alpha_{\text{1}}df_{it}+\alpha_{\text{2}}{Controls}_{it}+\mu_{t}+\lambda_{i}+\delta_{j}+\varepsilon_{it}\;$$ (2)

Among them, $M_{it}$ is the mechanism variable, which includes three variables: financing constraint (*sa*), environmental responsibility (*er*), and corporate reputation (*cr*). The meanings of the other variables are consistent with Model (1).

#### 4.4.1. Financing constraints alleviation effect.

To examine whether digital finance drives corporate climate risk information disclosure by alleviating financing constraints, this study employs the SA index developed by Hadlock et al. [53] to measure corporate financing constraints. Compared to the KZ and WW indices, which are potentially susceptible to endogeneity-driven measurement bias, the SA index is constructed based on two largely time-invariant and highly exogenous variables, namely firm size and age, using the formula: SA = −0.737 × Size + 0.043 × Size² - 0.040 × Age, where *Size* represents the natural logarithm of a firm’s total assets, *Age* denotes the number of a firm’s operating years, and a larger absolute value of the SA index (a negative-valued index) indicates more severe financing constraints. It has been widely adopted in relevant studies [54]. Column (1) of Table 8 reports the mechanism test results for the alleviation of financing constraints, showing that the digital finance coefficient is significantly negative. This implies that digital finance alleviates financing constraints faced by firms, thereby enabling them to allocate funds to climate risk management and effectively improve the completeness and timeliness of climate risk information disclosure. These findings validate the existence of the “financing constraints alleviation” mechanism.

**Table 8:** The results of mechanism tests.

Variable	(1)	(2)	(3)
	sa	er	cr
df	−0.073***	37.752***	5.253***
	(0.025)	(6.314)	(1.988)
lev	−0.019***	−1.293	1.698***
	(0.005)	(1.002)	(0.316)
curr	0.002***	−0.131***	−0.032**
	(0.000)	(0.049)	(0.014)
opm	−0.001	0.074	−0.013
	(0.001)	(0.135)	(0.062)
tagr	−0.003***	0.078	−0.075
	(0.001)	(0.108)	(0.049)
ins	−0.000	−1.397***	−0.099
	(0.002)	(0.445)	(0.182)
pd	0.021	−18.585***	0.636
	(0.015)	(3.537)	(1.264)
road	0.012	−0.417	−2.401***
	(0.011)	(3.339)	(0.925)
phone	0.000	0.089*	−0.040**
	(0.000)	(0.049)	(0.016)
Constant	−3.499***	−187.736***	−20.373*
	(0.138)	(35.377)	(11.193)
Firm FE	Y	Y	Y
Year FE	Y	Y	Y
Industry FE	Y	Y	Y
Observations	26,365	26,365	26,365
R-squared	0.984	0.723	0.840

Note: ***, **, and * represent statistical significance levels of 1%, 5%, and 10%, respectively. Robust standard errors are reported in parentheses.

#### 4.4.2. Environmental responsibility fulfillment effect.

To examine whether digital finance drives corporate climate risk information disclosure by reinforcing corporate environmental responsibility, this study employs the environmental sub-score in the ESG ratings of listed companies as a proxy for their ability to fulfill environmental responsibility. This metric encompasses multidimensional information, including pollution control, environmental management, and resource consumption, providing a comprehensive measure of a firm’s environmental responsibility. The higher this indicator, the stronger a firm’s ability to undertake environmental responsibility. Column (2) of Table 8 reports the results of the mechanism test for the enhanced effect of corporate environmental responsibility. The estimated coefficient for digital finance is significantly positive, indicating that digital finance can strengthen corporate awareness of environmental responsibilities. This heightened awareness motivates firms to prioritize and actively fulfill the responsibility of climate risk information disclosure. Thus, the “environmental responsibility reinforcement” mechanism is validated.

#### 4.4.3. Reputation incentive effect.

To test the mechanism of the reputational incentive effect, this study follows the approach of Zhou et al. [55] and employs the network search index for listed companies, published by the China Research Data Service Platform (CNRDS), to measure corporate reputation. The index captures the active attention and informational “connectedness” that the public and investors devote to a firm. A higher value indicates greater visibility within the social network, stronger digital connectivity with investors, heavier monitoring pressure, and consequently, a more pronounced reputational effect. Column (3) of Table 8 reports the results of the mechanism test for the reputational incentive effect, with the estimated coefficient on digital finance being significantly positive. This finding suggests that the development of digital finance enhances corporate reputation by raising a firm’s visibility in social networks, thereby incentivizing firms to disclose climate risk information in order to meet market expectations and consolidate their reputational advantage. Thus, the “reputational incentive effect” is confirmed.

To summarize, the results above confirm that digital finance drives firms to disclose climate risk information through three channels: alleviating financing constraints, strengthening environmental responsibility, and amplifying reputational incentives, thus verifying Hypothesis H2.

## 5. Further analysis

### 5.1. The moderating effect of institutional pressure

The theory of “pollution game” deeply reflects the “prisoner’s dilemma” in environmental governance, that is, in an environment lacking regulation, enterprises would rather pollute than increase environmental investment to achieve profit maximization. In order to avoid “the tragedy of the commons”, institutional constraints on enterprises are indispensable. The new institutionalism theory holds that the institutions influencing corporate behavior encompass not only formal institutions constructed by the government, which define behavioral boundaries through mandatory mechanisms such as legal regulations and administrative directives, but also informal institutional networks shaped by social actors such as social media and the public, which exert influence through invisible constraints like cultural cognition, moral norms, and value concepts. Formal and informal institutions are intertwined, constraining social subjects through rigid regulations, and promoting their behavior to meet expectations through flexible infiltration. Based on this framework, we attempt to explore how institutional pressure, across formal and informal dimensions, moderates the effect of digital finance on corporate climate risk information disclosure. Accordingly, an interaction term between institutional pressure and digital finance is incorporated into Model (1) to construct Model (3):


$$Y_{it}=\alpha_{\text{0}}+\alpha_{\text{1}}df_{it}+\alpha_{\text{2}}{institution}_{it}+\alpha_{\text{3}}df_{it}*{institution}_{it}+\alpha_{\text{4}}{Controls}_{it}+\mu_{t}+\lambda_{i}+\delta_{j}+\varepsilon_{it}\;$$ (3)

Among them, ${institution}_{it}$ represents institutional pressure, including formal and informal institutional pressures, while the meanings of other variables remain consistent with Model (1). In Model (3), the key parameter is $\alpha_{\text{3}}$. A significantly positive $\alpha_{\text{3}}$ would indicate that institutional pressure positively moderates the relationship between digital finance and corporate climate risk disclosure—that is, it amplifies digital finance’s facilitative effect on disclosure practices.

#### 5.1.1. Formal institutional pressure.

The government’s concern about the environment is typically reflected in policy formulation, regulatory stringency, and environmental governance goals. Essentially, it can directly influence corporate behavior through formal channels such as laws and regulations, administrative orders, fiscal incentives, or penalties, which conform to the core characteristics of formal institutional pressure. On the one hand, the policy pressure stemming from government environmental concern will compel firms to comply with environmental regulations and demonstrate their compliance through information disclosure. On the other hand, firms’ reliance on government-provided resources, such as environmental subsidies, pollution permits, and fiscal incentives, may motivate them to implement corresponding information disclosure policies. Therefore, we hypothesize that a higher level of government environmental concern enhances the positive effect of digital finance development on corporate climate risk information disclosure. To verify this hypothesis, we extract keyword frequencies related to six institutional environmental goals: “protecting and improving the environment”, “preventing and controlling pollution and other public hazards”, “resource conservation”, “coordinating development and environmental governance”, “promoting ecological civilization construction”, and “promoting sustainable economic and social development” from the work reports of various city governments to construct a government environmental concern index (*gec*). Subsequently, regression analysis is conducted using Model (3). As shown in column (1) of Table 9, the estimated coefficient of digital finance is significantly positive, and the coefficient of the interaction term (df * gec) between digital finance and government environmental concern is significantly positive. This indicates that government environmental concern can exert a policy signal-strengthening effect to enhance the positive effect of digital finance development on corporate climate risk information disclosure, thereby supporting the hypothesis previously mentioned.

**Table 9:** The results of regulatory tests.

	gc	media
Variable	(1)	(2)
	CRID	CRID
df	0.522***	0.509***
	(0.138)	(0.137)
ec	0.005	
	(0.004)	
df * ec	0.056**	
	(0.025)	
media		0.042
		(0.058)
df * media		0.604**
		(0.250)
lev	0.085***	0.086***
	(0.024)	(0.024)
curr	−0.002**	−0.002**
	(0.001)	(0.001)
opm	0.005	0.005
	(0.003)	(0.003)
tagr	0.011***	0.011***
	(0.004)	(0.004)
ins	0.011	0.010
	(0.010)	(0.010)
pd	−0.274***	−0.285***
	(0.076)	(0.076)
road	0.032	0.033
	(0.068)	(0.067)
phone	−0.002	−0.001
	(0.001)	(0.001)
Constant	−2.022***	−1.943**
	(0.772)	(0.769)
Firm FE	Y	Y
Year FE	Y	Y
Industry FE	Y	Y
Observations	26,365	26,365
R-squared	0.891	0.891

Note: ***, **, and * represent statistical significance levels of 1%, 5%, and 10%, respectively. Robust standard errors are reported in parentheses.

#### 5.1.2. Informal institutional pressure.

Cutting-edge research in socio-economics posits that social network structures profoundly influence the efficiency of information dissemination and the direction of capital allocation [56,57]. In this framework, the media, as the most critical information node and network amplifier in the modern information society, with their attention to firms constitute a central source of external informal institutional pressure on firms. It exerts social pressure on firms through flexible channels like information dissemination and public opinion oversight. Essentially, it accelerates the diffusion, interpretation, and capital pricing of information through vast social networks, thereby reshaping firms’ information environments and financing ecosystems and compelling firms to respond to social expectations [58]. Specifically, digital finance platforms (e.g., information modules embedded in mobile payment apps, social investment communities) interact and couple with media networks, which can significantly enhance the coverage and dissemination speed of climate risk information among investor communities [56]. For instance, after climate risk management measures disclosed by firms are reported by financial media, they can quickly trigger interactive discussions among young individual investors through “investor discussion forums” on digital finance platforms. This efficient information diffusion mechanism enables firms’ disclosure behaviors to be directly and rapidly converted into substantial reputational assets. Meanwhile, Social Connectedness has also been proven to have a significant impact on firms’ capital acquisition [43,57]. Especially in the highly interconnected environment built by digital financial tools, signals transmitted by media reports can enhance trust and “digital connectivity” between firms and investors (especially groups closely connected to digital platforms), further accelerating the widespread dissemination and discussion of firm information within investor communities, social media networks, and regional social connections. This social network-based dissemination mechanism can not only instantly amplify firms’ positive reputations but also quickly trigger trust crises in the event of environmental scandals, leading to the rapid withdrawal of social capital (e.g., investors’ concentrated stock sell-offs). Thus, firms are more inclined to proactively increase climate risk information disclosure to maintain social legitimacy, thereby avoiding potential losses caused by negative media reports [59]. Based on this, we hypothesize that when media attention to firms is high, the promoting effect of digital finance development on corporate climate risk information disclosure may be stronger. To verify this hypothesis, we measure media attention (*media*) by counting the total number of news items about companies published in financial newspapers and online financial news platforms, aiming to capture the information diffusion effect of such social networks. The data are sourced from the Financial News Database of Chinese Listed Companies (CFND). The regression results, shown in column (2) of Table 9, indicate that the estimated coefficient on digital finance is significantly positive, and the estimated coefficient on the interaction term between digital finance and media attention (df * media) is also significantly positive. This suggests that as amplifiers of social networks, media can strengthen information dissemination and capital connectivity effects, enabling digital finance-driven disclosure behaviors to be efficiently converted into firms’ reputational advantages and financing convenience, thereby significantly enhancing the incentive effect of digital finance on corporate climate risk information disclosure. Thus, our theoretical conjecture is supported.

### 5.2. Heterogeneity test

The baseline regressions presented above reveal a driving effect of digital finance on corporate climate risk information disclosure. However, a more profound question arises: Is this driving effect universal, or does it exhibit significant boundaries contingent upon the macro-environment and inherent firm characteristics? Drawing on the core view of neo-institutionalism that organizational behavior is jointly shaped by internal and external contexts, this section systematically examines the heterogeneity of how digital finance influences corporate climate risk information disclosure. We posit that digital finance does not operate passively within a static environment. Rather, its driving effect on corporate climate risk information disclosure is deeply embedded in the external foundational conditions of a firm’s region (e.g., rule of law environment, digital economy policies) and is simultaneously constrained by the firm’s intrinsic core attributes (e.g., executives’ overseas backgrounds, life cycle stage, and equity nature). This analysis aims to incorporate more contextual elements into the “digital finance-corporate behavior” research paradigm, thereby illuminating both the applicability and the boundary conditions of its governance effectiveness within the Chinese context. Ultimately, it seeks to provide a precise theoretical foundation and practical guidance for designing differentiated policies tailored to various types of regions and firms.

#### 5.2.1. Heterogeneity of rule of law environment.

The rule of law environment may introduce heterogeneity in the impact of digital finance on corporate climate risk information disclosure. The rule of law environment of a country or region plays a pivotal role in the corporate information disclosure quality [60]. Theoretically, in regions with stronger rule of law environments, laws and regulations are relatively sound and more strictly enforced, which can effectively supervise the information disclosure behavior of firms, ensuring that they disclose climate risk information truthfully, accurately, and completely. In contrast, in regions with weaker rule of law environments, the enforcement of laws and regulations may be insufficient, resulting in weaker constraints on corporate information disclosure. We therefore conjecture that the positive impact of digital finance on the quality of climate risk information disclosure may be stronger in regions with more robust rule of law environments. To test this hypothesis, we employ the sub-index of the rule of law environment from the *China Urban Business Environment Database* to measure the quality of the rule of law environment in each city. The research sample is then divided into higher- and lower-level rule of law environment groups based on the median value, and subgroup regression analyses are performed. As shown in columns (1) and (2) of Table 10, the estimated coefficient on digital finance is significantly positive in the subsample with higher-level rule of law environment but statistically insignificant in the subsample with lower-level rule of law environment. This indicates that digital finance more effectively promotes corporate climate risk information disclosure in cities with stronger rule of law environments than in those with weaker rule of law environments. This can be attributed to the following reasons. A sound rule of law environment typically features well-defined property rights protection and contract enforcement mechanisms, which bolster corporate confidence in making long-term reputational investments. In such contexts, firms are more inclined to view high-quality climate risk information disclosure as a strategic move to gain market trust [61]. Simultaneously, robust judicial oversight and the deterrent effect of law enforcement, coupled with the in-depth information scrutiny and risk identification capabilities of digital finance, jointly increase the risks and costs associated with false disclosure. This compels firms to disclose climate risk information more prudently and truthfully. In contrast, in regions with a weak rule of law, although digital finance provides technical convenience and financing incentives, the absence of effective legal constraints as a foundational guarantee hinders the full realization of its positive effect on corporate climate risk information disclosure.

**Table 10:** The results of heterogeneity of regional external environment.

	rule of law environment		digital economy policy supply	
Variable	(1)	(2)	(3)	(4)
	higher	lower	higher	lower
df	0.666**	0.080	0.981***	0.177
	(0.317)	(0.179)	(0.268)	(0.198)
lev	0.053	0.098***	0.064*	0.120***
	(0.037)	(0.034)	(0.038)	(0.036)
curr	−0.001	−0.002*	−0.003	−0.001
	(0.001)	(0.001)	(0.002)	(0.001)
opm	0.004	0.000	0.008*	0.008***
	(0.003)	(0.005)	(0.004)	(0.003)
tagr	0.013**	0.010***	0.010*	0.015***
	(0.006)	(0.004)	(0.006)	(0.005)
ins	0.035**	0.000	0.022	0.024
	(0.015)	(0.017)	(0.019)	(0.015)
pd	−0.245**	−0.571***	−0.360***	−0.147
	(0.108)	(0.167)	(0.114)	(0.575)
road	−0.317*	0.118	−0.025	0.107
	(0.183)	(0.086)	(0.126)	(0.093)
phone	−0.003**	0.010**	−0.002*	−0.001
	(0.001)	(0.004)	(0.001)	(0.003)
Constant	−2.807	0.381	−4.567***	−0.148
	(1.795)	(0.988)	(1.506)	(1.107)
Firm FE	Y	Y	Y	Y
Year FE	Y	Y	Y	Y
Industry FE	Y	Y	Y	Y
Observations	12,973	12,933	12,771	12,428
R-squared	0.911	0.895	0.907	0.897

Note: ***, **, and * represent statistical significance levels of 1%, 5%, and 10%, respectively. Robust standard errors are reported in parentheses.

#### 5.2.2. Heterogeneity of digital economy policy supply.

As a key institutional underpinning for the development of digital technologies, digital economy policies may profoundly influence the evolution of digital finance and corporate strategic choices by shaping and expanding the regulatory frameworks and resource networks essential for technological application. Based on this logic, we conjecture that the intensity of digital economy policy supply may affect the relationship between digital finance and corporate climate risk information disclosure. To test this proposition, following the approach of Tao and Ding [62], we use the frequency of keywords related to “digital economy” in various city government work reports to measure the supply intensity of digital economy policies in each city. The sample is then divided into high- and low-policy-supply groups based on the median value. The regression results, presented in columns (3) and (4) of Table 10, indicate that the estimated coefficient on digital finance is significantly positive in the subsample with higher-intensity digital economy policy supply, while it is statistically insignificant in the subsample with lower-intensity policy supply. This finding strongly supports the view that the higher the level of digital economy policy supply, the stronger the positive effect of digital finance on corporate climate risk information disclosure. In other words, robust digital economy policy support helps create a favorable development environment for digital finance, thereby promoting the level of corporate climate risk information disclosure. This heterogeneity can likely be attributed to the following reasons. Regions with a high level of digital economy policy supply typically feature more developed data factor markets, more pervasive digital infrastructure, and a stronger culture of societal innovation. These factors significantly lower the technical barriers and costs for firms to access and utilize digital financial tools. Concurrently, clear policy direction itself acts as a powerful institutional signal. It not only raises market expectations regarding the value of digital finance development but also guides the strategic allocation of financial resources by outlining clear industrial development priorities, thereby creating a favorable environment for financial institutions to develop and promote climate-friendly financial products. The combined action of this policy-induced “resource effect” and “signal effect” provides crucial support for fully realizing the promotive effect of digital finance on corporate climate risk information disclosure. Conversely, in cities with low digital economy policy supply, weak technological foundations and lagging innovation awareness hinder the effective realization of digital finance’s advantages due to a lack of supporting systems, consequently dampening its incentive effect on corporate climate risk information disclosure.

#### 5.2.3. Heterogeneity of overseas backgrounds among executives.

Upper echelons theory holds that the background characteristics of managers profoundly influence the strategic choices of enterprises and are crucial to organizational behavior [63]. We speculate that executives’ overseas backgrounds may shape the relationship between digital finance and the level of corporate climate risk information disclosure. To further verify this conjecture, we employ whether the current directors, supervisors, and senior managers have overseas study or work experience as a proxy indicator for executives’ overseas backgrounds. The indicator is coded as 1 if any of them have such experience and 0 otherwise. The regression results are shown in columns (1) and (2) of Table 11. The estimated coefficient on digital finance is significantly positive in firms with executives with overseas experience, but insignificant in those without such experience. These findings suggest that digital finance plays a more pronounced role in driving corporate climate risk information disclosure in firms with overseas-background executives. This discrepancy may be attributed to the fact that executives with overseas backgrounds may strengthen the promoting effect of digital finance on corporate climate risk information disclosure through their cognitive frameworks, institutional adaptability, and technological receptiveness. On the one hand, executives with overseas backgrounds are more likely to view climate risk information disclosure as a strategic imperative rather than a compliance cost [64]. This cognitive advantage motivates their willingness to adopt digital financial tools to improve the quality of information disclosure in order to meet the expectations of global investors and regulatory agencies. On the other hand, executives with overseas experience usually possess advanced management philosophies and a stronger sense of corporate social responsibility [65,66]. By deeply internalizing the climate governance logic prevalent in developed economies, they are able to learn from and introduce international information disclosure practices, embedding them into local operations. Concurrently, they actively leverage green financing channels to secure the necessary funding for high-quality disclosure. In addition, overseas experience can also help executives accumulate broader perspectives on technological innovation and knowledge, enhancing their ability to absorb and apply technologies [67]. This technological acuity enables a more precise alignment between the functionalities of digital financial instruments and the demands of climate risk management, consequently leading to improved corporate climate risk information disclosure. In contrast, executives lacking overseas backgrounds may be constrained by limited exposure to relevant concepts and practical experience, which impedes their ability to identify and utilize digital financial tools to address climate risk management demands.

**Table 11:** The results of heterogeneity of internal characteristics of enterprises.

	overseas backgrounds		life cycle			equity nature	
Variable	(1)	(2)	(3)	(4)	(5)	(6)	(7)
	no	yes	growth-stage	mature-stage	decline-stage	SOEs	non- SOEs
df	0.286	0.790***	0.314	0.666***	−0.150	0.637**	0.540***
	(0.207)	(0.206)	(0.262)	(0.244)	(0.385)	(0.265)	(0.168)
lev	0.126***	0.010	0.197***	−0.093*	−0.012	0.105**	0.081***
	(0.038)	(0.032)	(0.048)	(0.055)	(0.046)	(0.048)	(0.029)
curr	−0.001	0.000	0.000	0.000	−0.005***	−0.005	−0.002**
	(0.001)	(0.001)	(0.002)	(0.002)	(0.002)	(0.004)	(0.001)
opm	0.006	0.002	0.012**	−0.025*	−0.005	0.008	0.004
	(0.004)	(0.004)	(0.006)	(0.015)	(0.005)	(0.009)	(0.003)
tagr	0.021***	0.007*	0.013**	−0.002	−0.012	0.024**	0.007**
	(0.005)	(0.004)	(0.005)	(0.014)	(0.020)	(0.010)	(0.003)
ins	−0.010	0.019	0.009	0.049**	−0.025	0.025	0.015
	(0.017)	(0.012)	(0.018)	(0.019)	(0.021)	(0.021)	(0.011)
pd	−0.154	−0.302***	−0.268**	−0.358***	−0.064	−0.177	−0.206***
	(0.140)	(0.083)	(0.132)	(0.120)	(0.104)	(0.158)	(0.071)
road	−0.019	0.050	0.002	−0.014	0.081	−0.036	0.059
	(0.116)	(0.090)	(0.132)	(0.126)	(0.161)	(0.145)	(0.078)
phone	−0.003*	−0.001	0.000	−0.002	0.001	−0.004*	−0.002*
	(0.002)	(0.001)	(0.002)	(0.002)	(0.002)	(0.002)	(0.001)
Constant	−0.682	−3.504***	−0.800	−2.821**	1.669	−2.662*	−2.126**
	(1.162)	(1.154)	(1.459)	(1.372)	(2.156)	(1.493)	(0.942)
Firm FE	Y	Y	Y	Y	Y	Y	Y
Year FE	Y	Y	Y	Y	Y	Y	Y
Industry FE	Y	Y	Y	Y	Y	Y	Y
Observations	10,270	15,477	10,002	8,458	4,541	8,071	18,231
R-squared	0.909	0.904	0.916	0.912	0.906	0.910	0.885

Note: ***, **, and * represent statistical significance levels of 1%, 5%, and 10%, respectively. Robust standard errors are reported in parentheses.

#### 5.2.4. Heterogeneity of life cycle.

Corporate life cycle theory posits that firms exhibit distinct strategic priorities, resource endowments, and risk perceptions at different developmental stages. Accordingly, we hypothesize that the impact of digital finance on the disclosure of climate risk information by firms may differ across different corporate life cycle stages. To test this conjecture, following the approach of Dickinson [68], we use the cash flow pattern method to reflect the different life cycle stages of firms based on the positive and negative combinations of net cash flows from operations, investments, and financing activities. The sample is then divided into growth-, mature-, and decline- stage firm subsamples. The results, shown in columns (3) to (5) of Table 11, reveal that the estimated coefficient on digital finance is significantly positive in the mature-stage subsample, while it is statistically insignificant in the growth- and decline-stage subsamples. These results indicate that the positive effect of digital finance on corporate climate risk information disclosure is most prominent during the mature stage of firms. This heterogeneity can be explained as follows. During the growth stage, firms face severe resource constraints, which compel them to focus their strategic priorities on survival activities such as business expansion, capacity building, and market share competition. Consequently, climate management is often marginalized as a non-urgent expenditure in resource allocation. When firms enter the maturity stage, their business model and organizational structure become more established. Typically characterized by stable cash flows, mature organizational frameworks, and specialized teams, mature firms possess the necessary resource base to fully leverage digital finance for enhancing climate risk disclosure. Furthermore, having accumulated knowledge and experience during the growth phase, management is better equipped to integrate climate risk management into the strategic governance system, particularly in response to heightened demands for transparency from investors and regulators. Their perception of digital finance also evolves from viewing it as a mere operational “tool” to recognizing its potential for “strategic empowerment.” Consequently, they become more adept at utilizing digital financial instruments to improve the quality of climate risk disclosure, thereby maintaining market reputation and mitigating potential risks. However, when firms enter the decline stage, severe revenue declines compel management to cut non-core expenses, such as technology upgrades and climate risk management, to delay further deterioration. Such strategic contraction measures fundamentally impede the driving effect of digital finance on climate risk governance.

#### 5.2.5. Heterogeneity of equity nature.

Equity nature is a fundamental dimension for understanding behavioral heterogeneity among Chinese firms. There exist significant differences between state-owned enterprises (SOEs) and non-state-owned enterprises (non-SOEs) in terms of resource access, objective functions, and incentive mechanisms. We therefore hypothesize that the effect of digital finance on climate risk information disclosure may vary with firms’ equity nature. To explore this, the research sample is divided into SOE and non-SOE subsamples according to equity nature and subgroup regressions are performed. The estimated results, presented in columns (6) and (7) of Table 11, show that the estimated coefficient on digital finance is significantly positive at the 5% level in the SOE subsample, while it is significantly positive at the 1% level in the non-SOE subsample. This implies that, statistically speaking, digital finance exerts a stronger promotive effect on climate risk information disclosure in non-SOEs compared to SOEs. This divergence may stem from the following reasons. Driven by market survival logic and subject to intense competition, non-SOEs are highly sensitive to changes in capital costs and market signals. They are more inclined to proactively leverage digital financial tools to improve the quality of climate risk information disclosure, thereby sending positive signals to the market with the aim of securing green financing and attracting investor attention. This proactive stance ultimately amplifies the governance effect of digital finance. In contrast, as backbone entities entrusted with significant national economic responsibilities, SOEs benefit from government credit endorsements and policy protections, which diversify their financing channels and consequently weaken the financing incentives provided by digital finance. Meanwhile, climate risk information disclosure in SOEs is often subject to rigid institutional constraints such as environmental inspections and administrative assessments. This implies that their disclosure behavior may be primarily regulation-driven, which could crowd out the marginal contribution of digital finance. In addition, the inherent risk-averse organizational culture within SOEs may further dilute the potential of digital finance in enhancing climate risk information disclosure. Therefore, digital finance acts as a critical enabler for non-SOEs, whereas it functions more as a supplementary tool for SOEs.

## 6. Conclusion, policy implications, and future research directions

Disclosing climate risk information is not only an imperative for addressing global climate change, but also an important strategic instrument for practicing the concept of sustainable development. This study integrates digital finance and corporate climate risk information disclosure within a unified analytical framework, using Chinese A-share listed companies from 2016 to 2023 as research samples, to investigate the impact of digital finance on corporate climate risk information disclosure. The findings indicate that digital finance can significantly facilitate the disclosure of climate risk information by firms. Mechanism analysis indicates that digital finance can drive such disclosure by alleviating financing constraints, strengthening environmental responsibility, and amplifying corporate reputation incentives. Further research indicates that institutional pressure can positively moderate the effect of digital finance on corporate climate risk information disclosure. In addition, the driving effect of digital finance on corporate climate risk information disclosure varies depending on the external environment and internal characteristics of the firms. Specifically, from an external perspective, the positive effect of digital finance on corporate climate risk information disclosure is more pronounced in regions with a sound rule of law and a high level of digital economy policy supply. Internally, the driving effect of digital finance on corporate climate risk information disclosure is more evident in firms with executives having overseas experience, mature-stage enterprises, and non-SOEs.

Based on the above research findings, this study proposes the following countermeasures and suggestions: (1) Encourage innovation in digital financial instruments. Accelerate the development of a climate-oriented digital financial product system and enhance the pricing power of the capital market for climate risks. For instance, financial institutions should be encouraged to leverage climate-related data in developing financial derivatives, establish special incentive funds for digital climate risk information disclosure, and offer financing incentives and policy support to firms with superior disclosure ratings. (2) Strengthen corporate emphasis on climate risk information disclosure. Further refine relevant laws and regulations, and incorporate the quality of climate risk information disclosure into green performance assessment indicators. Build a climate disclosure credit system that supports in-depth integration between disclosure quality and green credit limits. Integrate digital climate risk management into the entrepreneurial spirit training system to enhance corporate social responsibility awareness and comprehensively improve firms’ capacity to identify and address climate risks. (3) Implement targeted measures to ensure effective government-enterprise collaboration. On the one hand, regional governments should continuously upgrade their governance systems by advancing digital governance in regions with well-established legal frameworks and fostering innovation ecosystems among digital economy policy frontrunners. They should also actively establish demonstration zones for digital-driven climate innovation and management to extend climate risk management practices to regions with weaker rule of law environments and less robust policy frameworks. On the other hand, it is necessary to guide firms to appoint Chief Climate Officers who are specifically responsible for developing and executing strategies related to digital financial tool applications and climate risk information disclosure. At the same time, tailored climate risk disclosure strategies should be implemented for firms at different life cycle stages to reduce the costs of using digital financial tools for climate risk information disclosure and to improve both the efficiency and quality of such disclosure.

Although this study strives for rigor, it still has several limitations that simultaneously point to promising avenues for future inquiry. First, regarding the examination of transmission mechanisms, this study identifies and verifies three parallel pathways: financing constraint alleviation, enhanced environmental responsibility, and reputational incentives. However, corporate management practices suggest these mechanisms are not isolated but may exhibit complex synergistic or sequential effects. For instance, financial resources freed through digital finance’s alleviation of financing constraints may provide the material foundation for firms to fulfill stricter environmental responsibilities, such as investing in emission-reduction technologies, which in turn could further strengthen their environmental reputation, creating a self-reinforcing cycle of “resources-responsibility-reputation.” Conversely, reputational damage arising from environmental negligence may exacerbate corporate financing difficulties. While our empirical design primarily tests the existence of these core pathways, it does not delve into the interrelationships and coupling patterns among them. Future research could employ structural equation modeling or other sophisticated analytical frameworks to elucidate whether these pathways complement, substitute, or dominate one another in the process through which digital finance influences corporate environmental behavior. Second, in terms of contextual factors, although this study introduces a socio-economic perspective and makes an initial attempt to proxy “social connectivity” and “online information diffusion” by using network search indices and media coverage, data limitations prevent us from constructing finer measures such as “the intensity of firm–investor interactions on social media” or “region-based social-network density.” Consequently, we cannot fully capture how digital finance reshapes “micro-level social connections.” Future studies could draw on methodologies employed by Kuchler et al. (2022) and Rehbein & Rother (2025), leveraging interaction data from platforms like Xueqiu or East Money’s stock forums, or constructing detailed social connection network maps based on geographic big data. Such approaches could more directly reveal how digital finance influences corporate climate risk disclosure by reshaping micro-level social connections, thereby providing richer micro-level evidence for interdisciplinary research spanning socio-economics and corporate finance.

## Supporting information

S1 File Data. (XLSX)
